# The Safety and Effectiveness of Melphalan-Based Intra-Arterial Chemotherapy for Retinoblastoma: An Updated Single-Arm Systematic Review and Meta-Analysis

**DOI:** 10.1155/2022/3156503

**Published:** 2022-02-14

**Authors:** Yang Cao, Mi Zhou, Min Tian, Hong-bin Lv

**Affiliations:** ^1^Department of Ophthalmology, Affiliated Hospital of Southwest Medical University, Luzhou 646000, China; ^2^Southwest Medical University, Luzhou 646000, China

## Abstract

Melphalan-based intra-arterial chemotherapy was considered an innovative treatment for retinoblastoma patients because high rates of globe salvage could be obtained. Now it has been widely applied for primary or secondary treatment of retinoblastoma. This meta-analysis summarizes the most up-to-date evidence regarding the safety and effectiveness of melphalan-based intra-arterial chemotherapy in the treatment of retinoblastoma. The authors searched PubMed, EMBASE, and the Web of Science electronic databases for studies investigating the safety and effectiveness of melphalan-based intra-arterial chemotherapy in the treatment of retinoblastoma. Studies reporting outcomes and complications of melphalan-based intra-arterial chemotherapy for the treatment of retinoblastoma patients would be included. A total of 33 observational studies that involved 1900 patients and 2336 eyes were included. The overall globe salvage rate was 79.6% (773/971 eyes, 0.74 [95% CI: 0.66, 0.80]) for patients treated with IAC as primary therapy in 28 studies. The overall globe salvage rate was 66.4% (923/1391 eyes, 0.68 [95% CI: 0.60, 0.76]) for patients treated with IAC as secondary therapy in 25 studies. The most common ocular complications were retinopathy (32%) and palpebral edema (29.7%). The most common systemic complications were nausea/vomiting (20.9%). The overall metastasis rate was 1.1% (21/1793 patients, 0.038 [95% CI: 0.020, 0.038]). Twenty-nine studies that involved 1783 patients reported the mortality and the overall mortality was 1.5% (26/1783 patients, 0.029 [95% CI: 0.020, 0.048]). Our meta-analysis showed that melphalan-based IAC treatment was an option for retinoblastoma patients with acceptable efficacy according to retrospective studies. Further high-quality randomized control trials are necessary to provide more accurate and reliable results.

## 1. Introduction

Retinoblastoma is the most common ocular malignancy in children, and the incidence is about 11 new cases per million individuals under 5 years old in Europe and the US [1, 2]. 75% of these patients will present with unilateral disease, with a median age peak of 2 to 3 years [1, 3]. Enucleation, systemic chemotherapy, radiotherapy, and local therapies are considered standard treatment methods. However, in the past decade, intra-arterial chemotherapy (IAC) was used for improving tumor control and increasing globe salvage rates as a primary or secondary treatment [4].

IAC, a local administration method, importantly avoided several adverse reactions caused by systemic chemotherapy such as ototoxicity and neurotoxicity [5]. Before the application of IAC, nearly 80% of advanced patients would eventually be forced to choose enucleation [6]. In recent years, melphalan-based intra-arterial chemotherapy has been extensively applied for the treatment of retinoblastoma patients [7]. Other major combination chemotherapy drugs include topotecan, carboplatin, and methotrexate. Though an increasing number of centers worldwide have adopted IAC, the optimal role for IAC is still undetermined.

Some previous systematic reviews have provided an extensive assessment of the evidence for IAC use in retinoblastoma [8, 9]. Since these studies, there have been several further studies published. In addition, the lack of randomized controlled trials makes the pivotal assessment of effectiveness and adverse reaction rates difficult. The authors conducted this systematic review and meta-analysis and provided an updated review of the IAC technique for the treatment of retinoblastoma patients.

## 2. Method

### 2.1. Inclusion Criteria

Studies that investigated the safety and effectiveness of melphalan-based intra-arterial chemotherapy for retinoblastoma and reported any of the following: globe salvage, ocular complications, systemic complications, metastasis, and death would be included.

### 2.2. Retrieval Strategy

This meta-analysis was performed according to the PRISMA (Preferred Reporting Items for Systematic Reviews and Meta-Analyses) recommendations. This study was not a human or animal experiment; thus, ethical approval was not necessary. PubMed, EMBASE, and the Web of Science electronic databases were searched with the terms “intra-arterial chemotherapy,” “intra-arterial therapy,” “melphalan,” and “retinoblastoma.” In addition, reference lists of the included studies were manually checked for potentially eligible studies, and Google Scholar search engines were used to find additional references. The last search was performed on October 8, 2021, without any restriction to the language of publication.

### 2.3. Literature Screening and Data Extraction

Two authors independently completed the literature screening and data extraction. The extracted general data included author, year, chemotherapy agents, follow-up, country of publication, and sample size. The main outcomes contained globe salvage, ocular complications, systemic complications, metastasis, and death. A third reviewer would be invited if there were any disputes.

### 2.4. Evaluation of Literature Quality

The methodological qualities of the non-RCTs were assessed independently by two authors using the Methodological Index for Non-Randomized Studies (MINORS) [10].

### 2.5. Statistical Analysis

Outcomes were estimated by calculating the pooled odds ratio (OR) (95% confidence intervals (CIs)) by RevMan software (version 5.1; Cochrane Collaboration, Copenhagen, Denmark). Heterogeneity was assessed by the *I*^2^ test. *I*^2^ < 50% suggests low heterogeneity. The analysis result of the single rate meta-analysis method was adopted (P2 and SE2 data), which requires effect size conversion [11]. Conversion of effect indicators: Pt = OR/(1+OR), 95% CI lower limit conversion: LL = LLOR/(1 + LLOR), and 95% CI upper limit conversion: UL = ULOR/(1 + ULOR).

## 3. Results

### 3.1. Search Results and Characteristics of Included Studies

**Figure 1:**
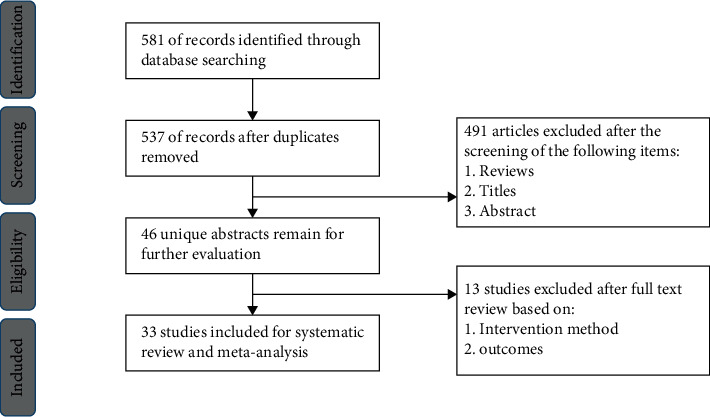
Flow diagram shows the process of literature selection.

A total of 581 potential articles were initially identified through database searches on 8 October 2021. A total of 537 studies were considered potentially eligible for further assessment after duplicates were removed. Finally, 33 observational studies [5, 11–42] that involved a total of 1900 patients and 2336 eyes published between 2011 and 2021 met the inclusion criteria and were included in this meta-analysis after a full-text review. All these studies reported indications for IAC as primary or secondary.  Figure 1 shows the literature selection process.  Table 1 summarizes the details of the included studies.

### 3.2. Literature Quality

All studies were assessed using the MINORS score (Table 2). All included studies scored 13–14. Due to the lack of a control group, the risk of bias was found in all the studies, and this was moderate throughout.

## 4. Outcomes

### 4.1. Globe Salvage

**Figure 2:**
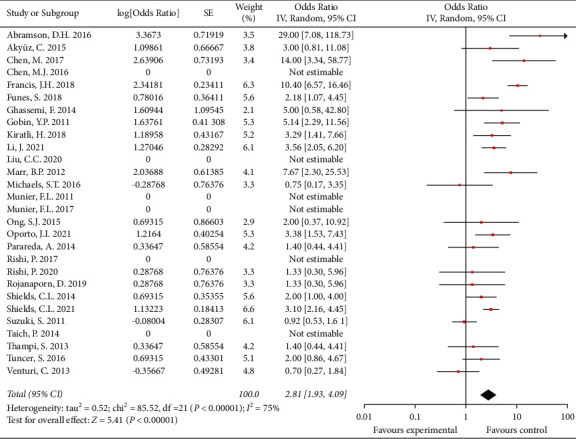
The overall globe salvage for patients treated with IAC as primary therapy.

**Figure 3:**
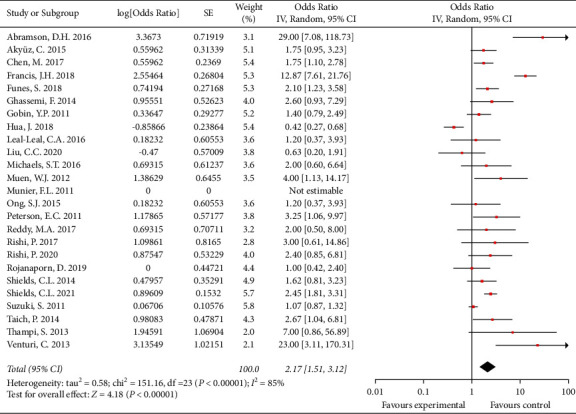
The overall effect size of globe salvage for patients treated with IAC as secondary therapy.

Thirty-three studies that involved 1900 patients and 2336 eyes reported globe salvage rates of 30% to 100%. The overall globe salvage rate was 79.6% (773/971 eyes) for patients treated with IAC as primary therapy in 28 studies. After pooling single-arm studies, the overall effect size of the proportion of globe salvage was 0.74 (95% CI: 0.66, 0.80) (Figure 2). The overall globe salvage rate was 66.4% (923/1391 eyes) for patients treated with IAC as secondary therapy in 25 studies. After pooling single-arm studies, the overall effect size of the proportion of globe salvage was 0.68 (95% CI: 0.60, 0.76) (Figure 3).

### 4.2. Ocular Complications

Ocular complications are described in Table 3. The most common ocular complications were retinopathy, with 8 events of 25 eyes and 25 patients (32%); palpebral edema, with 22 events of 74 eyes and 68 patients (29.7%); choroidal occlusion, with 5 events of 25 eyes and 21 patients (20%); and retinal detachment, with 28 events of 158 eyes and 148 patients (17.7%).

### 4.3. Systemic Complications

Systemic complications are described in Table 3. The most common systemic complications were nausea/vomiting, with 115 events of 549 patients (20.9%); cardiorespiratory disturbances, with 4 events of 25 patients (16%); and neutropenia, with 7 events of 64 patients (10.9%).

### 4.4. Metastasis

**Figure 4:**
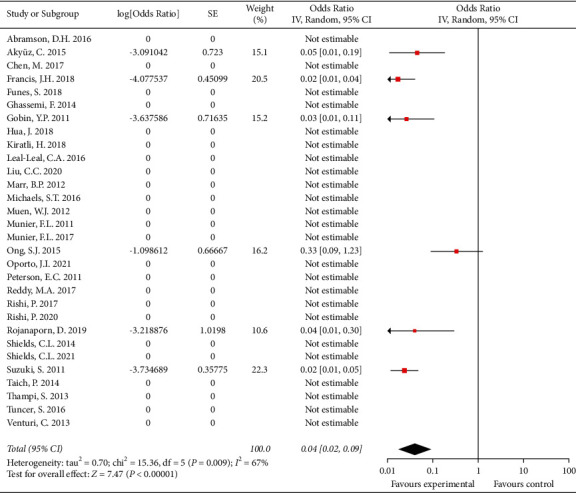
The overall effect size of the proportion of metastasis.

Thirty studies that involved 1793 patients reported the metastasis rate. Most patients in these studies did not have metastasis. The overall metastasis rate was 1.1% (21/1793 patients). After pooling single-arm studies, the overall effect size of the proportion of metastasis was 0.038 (95% CI: 0.020, 0.038) (Figure 4). Details are shown in Table 4.

### 4.5. Death

**Figure 5:**
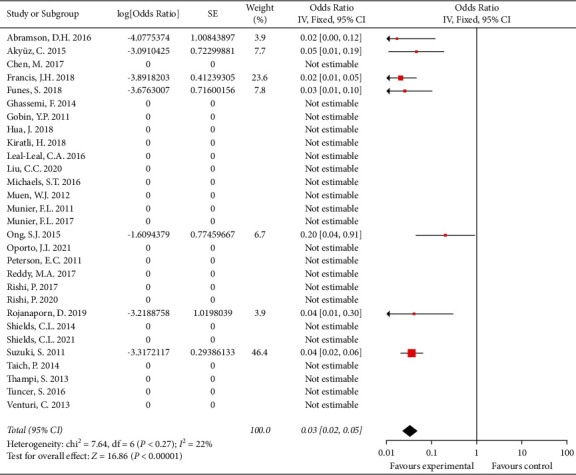
The overall effect size of the proportion of death.

Twenty-nine studies that involved 1783 patients reported the mortality, and the overall mortality was 1.5% (26/1783 patients). After pooling single-arm studies, the overall effect size of the proportion of metastasis was 0.029 (95% CI: 0.020, 0.048) (Figure 5). Details are shown in Table 4.

## 5. Discussion

Systemic chemotherapy remained the standard care for most advanced cancer patients, such as nonsmall cell lung cancer [43] and gastric cancer [44]. Systemic administration means that the drug will be acted on throughout the body, and it is more likely to have drug-related adverse effects.

A combination of intravenous chemotherapy with vincristine, etoposide, and carboplatin was the classical chemotherapy for retinoblastoma in the past [1]. Yamane et al. [45] first reported the selective ophthalmic arterial infusion of chemotherapy in 2004. Subsequently, despite the apparent technical challenge of effectively catheterizing a small vessel, this technique has become widely utilized. As a local administration method, intra-arterial chemotherapy has been performed in 26 countries worldwide in the last seven years [46]. Intra-arterial chemotherapy for retinoblastoma has been adopted as a first-line treatment option by numerous tertiary centers, and Ravindran et al. [9] performed a meta-analysis with 20 studies with a 35.6% globe salvage rate. However, various drugs were adopted in different studies. Besides, there have been several novel studies published afterward. Thus, it is necessary to update the results.

We conducted this meta-analysis with 33 studies involving a total of 1900 patients and 2336 eyes to evaluate melphalan-based intra-arterial chemotherapy for the management of retinoblastoma patients. IAC was used in all studies, and the chemotherapy drugs should include melphalan. The overall globe salvage rate was 79.6% for patients treated with IAC as primary therapy and 66.4% for patients treated with IAC as secondary therapy. These results were similar to a newly published systemic review performed by Runnels et al. [8], which included 24 studies. The globe salvage rate was lower than that reported by Ravindran et al. [9], which included 20 studies. However, IAC used by primary or secondary was not considered in that study. Periocular edema (10.5%) was the most common ocular complication reported in the systemic review performed by Runnels. However, the most common ocular complication in our study is retinopathy (32%), followed by palpebral edema (29.7%). Besides, we reported a lower rate of metastasis (1.1%) and death (1.5%).

### 5.1. Limitations of This Study

First, due to the lack of randomized controlled trials, we cannot perform this meta-analysis based on high-level studies. Second, several other chemotherapeutic regimens were included besides melphalan, though we have tried to limit the study to at least melphalan. This may still lead to a certain degree of heterogeneity. Third, little information was known about progression-free survival and disease control rates after IAC treatment, as these are important indicators of treatment effectiveness.

### 5.2. Conclusions

Our meta-analysis showed that melphalan-based IAC treatment was an option for retinoblastoma patients with acceptable efficacy according to retrospective studies. Further high-quality randomized control trials are necessary to provide more accurate and reliable results.

## Figures and Tables

**Table 1:** Characteristics of included studies.

Study	Chemotherapy agents	Number of eyes	Primary number of eyes	Secondary number of eyes	Follow-up duration (months)	County/region	Design
Abramson, et al. 2016	Melphalan, topotecan, carboplatin, and methotrexate	120	60	60	36.0	USA	Retrospective
Akyüz, et al. 2015	Melphalan	56	12	44	11.9	Turkey	Retrospective
Chen, et al. 2017	Melphalan, topotecan, and carboplatin	107	30	77	13.6^#^	China	Retrospective
Chen, et al. 2016	Melphalan, topotecan, and carboplatin	13	13	NA	28^#^	China	Retrospective
Francis, et al. 2018	Melphalan, topotecan, and carboplatin	436	228	208	23.6	USA	Retrospective
Funes, et al. 2018	Melphalan, topotecan, and carboplatin	97	35	62	48.7	Argentina	Retrospective
Ghassemi, et al. 2014	Melphalan, topotecan, and carboplatin	24	6	18	17	Iran	Retrospective
Gobin, et al. 2011	Melphalan, topotecan, carboplatin, and methotrexate	91	43	48	13.0	USA	Retrospective
Hua, et al. 2018	Melphalan and topotecan	84	0	84	14.2^#^	China	Retrospective
Kiratli, et al. 2018	Melphalan and topotecan	30	30	NA	4.0^#^	Turkey	Retrospective
Leal-Leal, et al. 2016	Melphalan and topotecan	11	0	11	14.3^#^	Mexico	Retrospective
Li, et al. 2021	Melphalan, topotecan, and carboplatin	73	NA	NA	7	China	Retrospective
Liu, et al. 2020	Melphalan, topotecan, and carboplatin	14	1	13	17.0	Malaysia	Retrospective
Marr, et al. 2012	Melphalan, topotecan, and carboplatin	26	26	NA	14^#^	USA	Retrospective
Michaels, et al. 2016	Melphalan, topotecan, and carboplatin	19	7	12	13.0	USA	Retrospective
Muen, et al. 2012	Melphalan	15	0	15	9	UK	Retrospective
Munier, et al. 2011	Melphalan	13	9	4	7.0	Switzerland	Retrospective
Munier, et al. 2017	Melphalan	25	25	NA	41.7^#^	Switzerland	Retrospective
Ong, et al. 2015	Melphalan	17	6	11	22	Taiwan	Retrospective
Oporto, et al. 2021	Melphalan and topotecan	35	NA	NA	36.5	Chile	Retrospective
Parareda, et al. 2014	Melphalan	12	12	NA	29.5	Spain	Prospective
Peterson, et al. 2011	Melphalan	17	0	17	8.6^#^	USA	Retrospective
Reddy, et al. 2017	Melphalan and topotecan	9	0	9	21.0	UK	Retrospective
Rishi, et al. 2017	Melphalan and topotecan	10	2	8	26.0	India	Retrospective
Rishi, et al. 2020	Melphalan and topotecan	24	7	17	28.6	India	Retrospective
Rojanaporn, et al. 2019	Melphalan, topotecan, and carboplatin	27	7	20	32^#^	Thailand	Retrospective
Shields, et al. 2014	Melphalan, topotecan, and carboplatin	70	36	34	19.0	USA	Retrospective
Shields, et al. 2021	Melphalan, topotecan, and carboplatin	341	160	207	NA	USA	Retrospective
Suzuki, et al. 2011	Melphalan	408	50	358	74.0	Japan	Retrospective
Taich, et al. 2014	Melphalan and topotecan	27	5	22	11.7	Argentina	Retrospective
Thampi, et al. 2013	Melphalan	20	12	8	15	USA	Retrospective
Tuncer, et al. 2016	Melphalan	24	24	NA	29	Turkey	Retrospective
Venturi, et al. 2013	Melphalan	41	17	24	13.0	Italy	Retrospective

Number^#^: median; NA: not available.

**Table 2:** MINORS appraisal scores for the included retrospective studies.

Study	Methodologic items^*∗*^												Total
	1	2	3	4	5	6	7	8	9	10	11	12	
Abramson, et al. 2016	2	2	0	2	0	2	2	0	0	2	0	2	14
Akyüz, et al. 2015	2	2	0	2	0	2	2	0	0	2	0	2	14
Chen, et al. 2017	2	2	0	2	0	2	1	0	0	2	0	2	13
Chen, et al. 2016	2	2	0	2	0	2	1	0	0	2	0	2	13
Francis, et al. 2018	2	2	0	2	0	2	1	0	0	2	0	2	13
Funes, et al. 2018	2	2	0	2	0	2	2	0	0	2	0	2	14
Ghassemi, et al. 2014	2	2	0	2	0	2	2	0	0	2	0	2	14
Gobin, et al. 2011	2	2	0	2	0	2	2	0	0	2	0	2	14
Hua, et al. 2018	2	2	0	2	0	2	2	0	0	2	0	2	14
Kiratli, et al. 2018	2	2	0	2	0	2	2	0	0	2	0	2	14
Leal-Leal, et al. 2016	2	2	0	2	0	2	2	0	0	2	0	2	14
Li, et al. 2021	2	2	0	2	0	2	2	0	0	2	0	2	14
Liu, et al. 2020	2	2	0	2	0	2	2	0	0	2	0	2	14
Marr, et al. 2012	2	2	0	2	0	2	2	0	0	2	0	2	14
Michaels, et al. 2016	2	2	0	2	0	2	2	0	0	2	0	2	14
Muen, et al. 2012	2	2	0	2	0	2	2	0	0	2	0	2	14
Munier, et al. 2011	2	2	0	2	0	2	2	0	0	2	0	2	14
Munier, et al. 2017	2	2	0	2	0	2	1	0	0	2	0	2	13
Ong, et al. 2015	2	2	0	2	0	2	1	0	0	2	0	2	13
Oporto, et al. 2021	2	2	0	2	0	2	2	0	0	2	0	2	14
Parareda, et al. 2014	2	2	0	2	0	2	2	0	0	2	0	2	14
Peterson, et al. 2011	2	2	0	2	0	2	2	0	0	2	0	2	14
Reddy, et al. 2017	2	2	0	2	0	2	2	0	0	2	0	2	14
Rishi, et al. 2017	2	2	0	2	0	2	1	0	0	2	0	2	13
Rishi, et al. 2020	2	2	0	2	0	2	1	0	0	2	0	2	13
Rojanaporn, et al. 2019	2	2	0	2	0	2	1	0	0	2	0	2	13
Shields, et al. 2014	2	2	0	2	0	2	2	0	0	2	0	2	14
Shields, et al. 2021	2	2	0	2	0	2	2	0	0	2	0	2	14
Suzuki, et al. 2011	2	2	0	2	0	2	2	0	0	2	0	2	14
Taich, et al. 2014	2	2	0	2	0	2	2	0	0	2	0	2	14
Thampi, et al. 2013	2	2	0	2	0	2	2	0	0	2	0	2	14
Tuncer, et al. 2016	2	2	0	2	0	2	2	0	0	2	0	2	14
Venturi, et al. 2013	2	2	0	2	0	2	2	0	0	2	0	2	14

^*∗*^Methodologic items: (1) a clearly stated aim; (2) inclusion of consecutive patients; (3) prospective collection of data; (4) endpoints appropriate to the aim of the study; (5) unbiased assessment of the study endpoint; (6) follow-up period appropriate to the aim of the study; (7) loss to follow-up, which is less than 5%; (8) prospective calculation of the study size; (9) an adequate control group; (10) contemporary groups; (11) baseline equivalence of groups; and (12) adequate statistical analyses. The items are scored as “0” (not reported), “1” (reported but inadequate), or “2” (reported and adequate).

**Table 3:** Complication.

Complications	No. of events	Total eyes	Rate	Total patients
*Ocular complications*				
Avascular retinopathy	5	158	0.032	137
Arteriolar sclerosis	2	12	0.167	11
Aseptic cellulitis	2	35	0.057	29
Cataract	12	201	0.060	165
Chorioretinal atrophy	31	626	0.050	535
Choroidal occlusion	5	25	0.200	21
Choroidal ischemia	7	341	0.021	313
Conjunctiva chemosis	1	14	0.071	14
Extraocular muscle paresis	0	24	0.000	22
Internal carotid artery occlusion	0	24	0.000	22
Loss of eyelashes	21	165	0.127	143
Multinucleated macrophages in choroid and retina	2	12	0.167	11
Neovascular glaucoma	1	26	0.038	24
Neovascularisation	55	366	0.150	338
Oculomotor nerve palsy	2	35	0.057	29
Ophthalmic artery occlusion	0	24	0.000	22
Occlusive vasculopathy	22	276	0.080	232
Optic nerve disorder	2	24	0.083	15
Ophthalmoplegia	10	123	0.081	121
Phthisis	7	132	0.053	112
Ptosis	25	366	0.068	330
Periocular edema	107	1019	0.105	829
Palpebral oedema	22	74	0.297	68
Palpebral erythema	1	25	0.040	25
Periorbital pigmentation	1	35	0.029	29
Retinopathy	8	25	0.320	25
Retinal atrophy	2	12	0.167	11
Retinal detachment	28	158	0.177	148
Retinal ischemia	13	341	0.038	313
Retinal artery precipitation	6	79	0.076	70
Strabismus	3	54	0.056	60
Vitreous hemorrhage	55	448	0.123	366
Vascular spasm	2	25	0.080	21

*Systemic complications*				
Anaphylaxis	3		0.039	77
Bronchospasm	34		0.062	549
Cardiorespiratory disturbances	4		0.160	25
Fever	47		0.081	579
Groin hematoma	1		0.067	15
Limb ischemia	0		0.000	349
Neutropenia	7		0.109	64
Nausea/vomiting	115		0.209	549
Stroke	2		0.002	846
Transfusion	1		0.001	680
Thromboembolism	0		0.000	14
Vascular dissection	0		0.000	313
Vasospasm	2		0.080	25

**Table 4:** Metastasis and death.

Study	Number of patients	Number of metastasis	Number of deaths
Abramson, et al. 2016	60	0	1
Akyüz, et al. 2015	46	2	2
Chen, et al. 2017	73	0	0
Chen, et al. 2016	10	0	NA
Francis, et al. 2018	300	5	6
Funes, et al. 2018	81	0	2
Ghassemi, et al. 2014	24	0	0
Gobin, et al. 2011	78	2	0
Hua, et al. 2018	62	0	0
Kiratli, et al. 2018	28	NA	0
Leal-Leal, et al. 2016	11	0	0
Li, et al. 2021	71	NA	NA
Liu, et al. 2020	14	0	0
Marr, et al. 2012	25	0	NA
Michaels, et al. 2016	17	0	0
Muen, et al. 2012	14	0	0
Munier, et al. 2011	13	0	0
Munier, et al. 2017	25	0	0
Ong, et al. 2015	12	3	2
Oporto, et al. 2021	29	0	0
Parareda, et al. 2014	11	NA	NA
Peterson, et al. 2011	15	0	0
Reddy, et al. 2017	9	0	0
Rishi, et al. 2017	10	0	0
Rishi, et al. 2020	15	0	0
Rojanaporn, et al. 2019	26	1	1
Shields, et al. 2014	67	0	0
Shields, et al. 2021	313	0	0
Suzuki, et al. 2011	343	8	12
Taich, et al. 2014	26	0	0
Thampi, et al. 2013	16	0	0
Tuncer, et al. 2016	22	0	0
Venturi, et al. 2013	34	0	0
Total (event)		21	26
Total (patients)		1793	1783
Rate		1.1%	1.5%

NA: not available.

## Data Availability

All data generated or analyzed during this study are included within the article.
